# Modifying Pharmacokinetic Properties of the Gram-Negative Bacteria Targeting Endolysin ML06 Without Affecting Antibacterial Activity

**DOI:** 10.3390/ijms26094376

**Published:** 2025-05-04

**Authors:** Nataliia P. Antonova, Sofia D. Abdullaeva, Daria V. Vasina, Igor V. Grigoriev, Evgeny V. Usachev, Olga V. Usacheva, Vladimir A. Gushchin

**Affiliations:** 1 N. F. Gamaleya National Research Centre for Epidemiology and Microbiology, Ministry of Health of the Russian Federation, 123098 Moscow, Russiad.v.vasina@gmail.com (D.V.V.); wowaniada@yandex.ru (V.A.G.); 2 Department of Medical Genetics, Federal State Autonomous Educational Institution of Higher Education I M Sechenov First Moscow State Medical University of the Ministry of Health of the Russian Federation (Sechenov University), 119991 Moscow, Russia

**Keywords:** Gram-negative bacteria, endolysin, albumin-binding domain, dimerization, antibacterial activity, western blot, serum stability, pharmacokinetics, intravenous administration

## Abstract

With the rise of antibiotic resistance, there is a need for innovative drugs with alternative mechanisms of action. Endolysins meet most of the requirements, but are limited for parenteral use due to their short blood circulation time. In this article, a number of modifications to the ML06-engineered, lysin-targeting Gram-negative bacteria are proposed to improve its pharmacokinetic parameters. Genetic modification with albumin-binding and dimerization domains ranging from 11–12 aa to 45 aa at both the C- and N-termini has resulted in six enzymes that do not exhibit critically reduced antibacterial properties in vitro, and in the case of the ABP1 modification, an improved antibacterial rate and spectra of enzymes. The ML06-ABP1, ML06-ABP2, and HDD-ML06 modifications also retained activity in blood serum and significantly increased serum stability. A pharmacokinetic study of the three modifications in mice showed that ML06-ABP2 and HDD-ML06 have a prolonged half-life compared to the ML06 half-life. In addition, the serum C_max_ concentration for HDD-ML06 (22.2 μg/mL) was significantly increased compared to ML06 (C_max_ < 5 μg/mL). Our results allow for a comparison of the different types of modifications that are useful in the development of parenteral antibacterials.

## 1. Introduction

The development of alternative antibacterial agents to combat multidrug-resistant bacteria is still an urgent task for the scientific community. Gram-negative pathogens of the ESKAPE group, such as Enterobacteriaceae, *Acinetobacter baumannii*, and *Pseudomonas aeruginosa*, are of particular interest because they are the most likely to acquire multiple resistance to antibiotics (including carbapenems and third-generation cephalosporins) and cause the most serious consequences of infection [1].

Among a number of alternative strategies [2], bacteriophage lytic enzymes (particularly endolysins) have emerged as an actively developing class of antibacterial compounds with potential for clinical use. Numerous studies are currently investigating the antibacterial properties of endolysins and their modified derivatives in vitro, their efficacy in animal infectious disease models, and preclinical safety [3,4,5,6,7]. For severe systemic infections, several endolysin-based antibacterials targeting drug-resistant *Staphylococcus aureus* are in various stages of clinical trials as an addition option to gold-standard antibiotics [8]. However, endolysins for the treatment of Gram-negative systemic bacterial infections are not sufficiently covered and there is no information on clinical trials of such enzymes to date.

Numerous studies have shown that lysins are able to act on bacteria regardless of pathogens’ antibiotic resistance, indicating that the treatment of life-threatening bacteremia caused by resistant strains may become one of the main uses of endolysin-based drugs as a “last resort” therapy. At the same time, when administered intravenously, endolysins, like other therapeutic protein molecules, are rapidly exposed to the immune system and blood proteases, filtered by the kidneys, and eliminated from the systemic circulation within 0.5–2 h [9]. This may not be sufficient to achieve antimicrobial activity. It is therefore necessary to improve their pharmacokinetic parameters in order to obtain effective substances.

Several approaches to modify the pharmacokinetic properties of protein drugs have been discussed in the literature. Attempts to increase the half-life of lytic enzymes have included PEGylation, glycosylation, depletion of T-cell epitopes, dimerization, and fusion with albumin-binding domains [10,11]. It has been shown that the PEGylation can inhibit enzymes targeting Gram-positive bacteria [12]. Conjugation with biomolecules, such as fatty acids, has led to a significant increase in protein half-life in mice (0.4 h vs. 1.5 h), improving animal survival and bacterial elimination in the *A. baumannii* bacteriemia model [13].

However, protein bioengineering offers the broadest opportunities to modify enzymes’ properties, and in the case of bacteriolytic enzymes, this strategy seems to be the most effective. For example, antistaphylococcal enzymes LysK and lysostaphin were fused to different types of albumin-binding domains (ABDs), and their half-lives were increased [14,15]. Also, dimerization strategy was successfully used for lysostaphin and antipneumococcal lysin Cpl-1 [16,17]. It should be noted that endolysins, especially in the case of Gram-negative bacteria-targeting enzymes, are small globular proteins with tightly packed domains, and even small changes in their structure can lead to a loss of functional activity or biotechnological properties. The efficacy of such modifications varies for proteins and only a few comprehensive studies have been carried out for endolysins [10].

In our study, the modified endolysin ML06 was chosen as the model enzyme. It is a chimeric lysin containing muramidase LysAp22 (lysozyme-like glycoside hydrolase) as an enzymatically active domain and a fragment of the SMAP-29 antimicrobial peptide for the enhancement of membranes’ penetrating properties. We have previously shown that this peptide allows several endolysins to enhance antibacterial activity under physiological conditions [18,19]. In this article, we investigate different modifications aimed to reduce the degradation and renal excretion of ML06 endolysin and compare their biotechnological, antibacterial, and pharmacokinetic properties. To date, this is the first study of the genetic engineering approaches to Gram-negative bacteria targeting endolysin in the context of pharmacokinetic improvement.

## 2. Results

### 2.1. Construction and Production of Modified Endolysins

In this article, two widely used protein engineering options (Table 1) were considered for increasing the half-life of the endolysin ML06. These included modifications with ABDs and enzyme dimerization.

The albumin-binding modifications allow the molecule to bind to the albumin in the blood, which in turn binds to the FcRn receptor in the endosomes and prevents the lysosomal degradation of the molecule [20]. In addition, these modifications do not increase the size of the molecule as much as the Fc region of human IgG1 antibodies (approximately 180 aa), so steric hindrances to the enzymatic action of the enzyme and the production of the endolysin’s inactive form are less likely. We have chosen albumin-binding peptides of 11 and 12 aa (ABP1 and ABP2) obtained via phage display of peptide libraries [21]. These peptides have a high affinity for albumins from different animal species (mouse, rat, rabbit, and human). Next, the 45 aa streptococcal protein G albumin-binding domain (StrepABD) was used, whose sequence was added to endolysin via a flexible linker [15].

Dimerization of the molecule increases the hydrodynamic size of the protein above the renal filtration threshold of 40–50 kDa, thereby increasing its half-life in the body [22]. The 45 aa homodimerization domain (HDD), which allows for the formation of antiparallel α-helices [16], is also introduced into the structure via flexible linkers.

Eight genetic constructs encoding modified variants of the endolysin ML06 were obtained in the expression vector pET42b (+), considering the possibility of each modification at the N- and C-termini of the polypeptide. The prokaryotic expression system (*E. coli* Rosetta) was used for heterologous recombinant expression of the proteins (Figure S1). The expression strains obtained were characterized by different biomass yields ranging from 1.8 to 4.7 g per 1 L of culture broth, and N-terminal modifications resulted in a 1.4–2.4-fold decrease in biomass yield in all cases (Table 2).

Two proteins with the albumin-binding peptide ABP2 at the N- and C-termini of lysin (ML06-ABP2 and ABP2-ML06) were shown to be expressed mostly in the soluble fraction as well as the parental ML06 (Table 2). The remaining six molecules were expressed completely in inclusion bodies. Moreover, we have not been able to pick up the conditions for the refolding of two proteins (ML06-StrepABD and ML06-HDD), both of which were long modifications (45 aa) with flexible linkers at the C-terminus. Although the biomass growth rates for modifications were comparable to or lower than for ML06, the formation of predominantly insoluble fractions indicated high rates of protein synthesis accompanied by insufficient chaperone folding of the tertiary structure. Alternatively, the toxicity of expression products to the host cell can be proposed to affect the cells’ viability.

**Figure 1:**
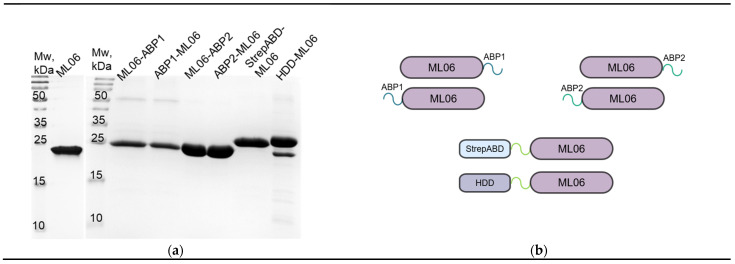
Modified endolysin variants obtained: (**a**) Electrophoregrams of the recombinant endolysin solutions; Mw, molecular weight marker. (**b**) Schematic representation of the enzymes.

The modified proteins were purified to >95% purity with several steps of FPLC (Figure 1a,b). The only exception was HDD-ML06 (>90%), which had an additional band of co-expressed admixture, which is proposed to be the truncated protein version without the homodimerization domain (ML06). The protein yields (Table 2) were also lower for modifications compared to ML06 (up to fivefold), and only StrepABD-ML06 showed an almost threefold increase in production, but was easily aggregated in solution, regardless of the concentration.

Thus, we have shown that modifying the endolysin sequence with additional peptides/domains significantly affects the recombinant expression of the protein, in most cases leading to reduced production by *E. coli* and the formation of insoluble proteins under standard induction conditions. The data also showed that the C-terminus modifications of lysin are relatively more toxic for the recombinant producer strain of *E. coli*, slowing its growth, while the growth of recombinant hosts of proteins with N-terminus modifications is comparable to that of the original enzyme producer.

### 2.2. Antibacterial Properties of the Modified Enzymes

**Figure 2:**
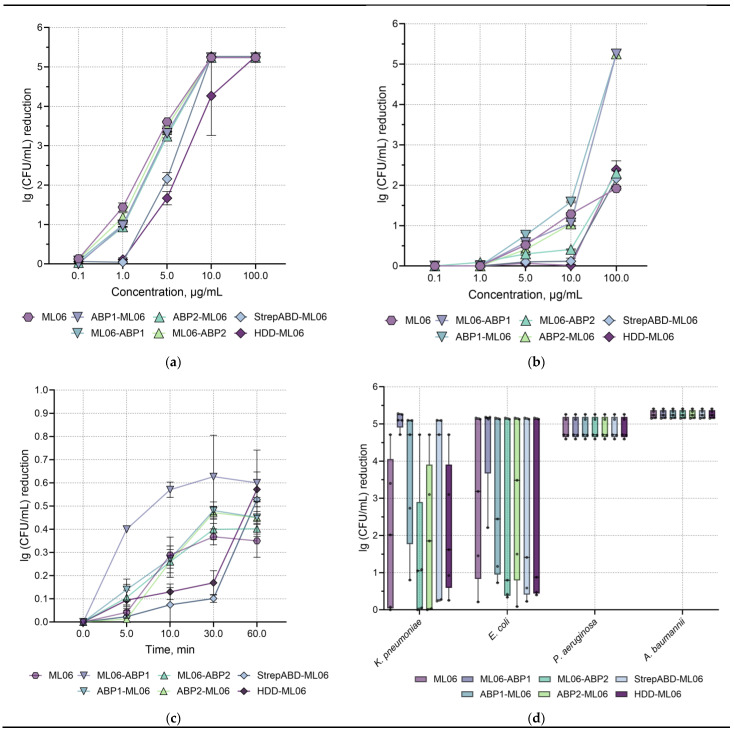
Antibacterial properties of the modifies endolysins: (**a**) Dose-dependent activity of the enzymes against exponentially growing and (**b**) stationary phase *A. baumannii* 50-16 (30 min incubation time). (**c**) Time–kill curve (0.5 μg/mL enzyme concentration, 1 μg/mL for StrepABD-ML06 and HDD-ML06). Data are shown as mean lg (CFU/mL) reduction relative to negative control ± standard deviation (SD). (**d**) Enzyme spectrum of action (100 μg/mL, 60 min incubation). Each dot indicates activity towards individual strains (lg (CFU/mL) reduction), n = 5 for each species; line; median; box; IQR whisker; min–max.

In vitro studies of the antibacterial activity of the modified endolysins were carried out in comparison with the parent ML06. Using the model *A. baumannii* strain, it was shown that the introduction of the small peptides ABP1 and ABP2 (11–12 aa) did not dramatically affect the dose-dependent antibacterial activity against exponentially growing bacterial cells after incubation for 30 min (Figure 2a). Modifications with a molecular weight of 45 aa (StrepABD-ML06 and HDD-ML06) significantly decreased the bacterial reduction by 0.96 and 0.86 lg (CFU/mL), respectively, at 1.0 µg/mL protein concentrations. The effective dose ED50 (Table 3) was reduced 1.2–2.4-fold for ABP1 and ABP2 modifications and 7.1- and 9.2-fold for StrepABD and HDD, respectively, compared to ML06.

Significant decreases of 0.66 lg, 0.95 lg, and 1.05 lg relative to ML06 (at 10.0 µg/mL concentration) were observed for the ML06-ABP2, StrepABD-ML06, and HDD-ML06 modifications in stationary-phase bacteria, which are characterized by increased resistance to the effects of antibacterials due to changes in their cell wall structure (Figure 2b). At the same time, the N-terminal modification of ML06 with albumin-binding peptides, ABP1-ML06 and ABP2-ML06, resulted in higher antibacterial activity (approximately by 3 lg (CFU/mL)) compared to other enzymes at a concentration of 100 µg/mL. The ED50 was slightly increased (1.1–1.3-fold) for both ABP1 modifications, reduced 1.1–1.7-fold for both ABP2modifications, 3.1-fold for StrepABD, and 4.8-fold for HDD compared to ML06 (Table 3).

Furthermore, when investigating the rate of enzyme action (Figure 2c), significant changes were observed in the time–kill curves: ML06 and ABP2-ML06 had a 5 min lag phase and reached a plateau of activity after 30 min of incubation; StrepABD-ML06 and HDD-ML06 had the longest lag phase of 30 min and reached maximum activity after 60 min of incubation, indicating that the highest-molecular-weight modifications result in slower activity. The ML06-ABP1, ABP1-ML06, and ML06-ABP2 modifications did not have a pronounced lag phase and acted from the first minutes of incubation, with the ML06-ABP1 enzyme showing the highest speed of action. Further spectra of action experiments were performed by incubating the proteins with bacteria for 60 min at a concentration of 100 µg/mL to equalize the conditions.

The study of the activity spectra of the modified endolysins (Figure 2d) against different types of Gram-negative bacterial strains (*P. aeruginosa*, *A. baumannii*, *K. pneumoniae*, and *E. coli*) showed that all the modified endolysins lysed ~5 lg (CFU/mL) of *P. aeruginosa* and *A. baumannii*. In Enterobacteriaceae, which are less susceptible to the action of lytic enzymes, modification with the albumin-binding peptide ABP1 significantly increased the activity of the molecules compared to the ML06 (by 1.6–2.1 lg). We also showed that C-terminal modifications via the ABP1 albumin-binding peptide resulted in higher activity than the same N-terminal modification: for *K. pneumoniae* and *E. coli*, activity was 1.4 and 1.7 lg (CFU/mL) higher, respectively. Modifications with the ABP2 peptide slightly reduced (by 0.6 lg) or did not affect the spectrum of activity. At the same time, the activity of the hybrid with the modification at the N-terminus was higher by 0.5 and 0.7 lg (CFU/mL), so no pronounced pattern regarding the site of modification via the ABP peptides was detected. The StrepABD-ML06 and HDD-ML06 modifications had increased (by 1.1 lg for StrepABD-ML06) or had no effect against *K. pneumoniae*, but slightly decreased activity against *E. coli* (by 0.5–0.6 lg).

To sum up, the introduction of modifications had no critical effect on the activity of the enzymes and in some cases showed positive effects. No general pattern between modifications could be identified when comparing enzymes with albumin-binding domains hybridized at the N- and C-termini of the ML06 molecule. At the same time, longer modifications (StrepABD and HDD) affected almost all aspects of the enzyme’s activity, but did not alter its antibacterial spectrum, and the effects were neutralized at higher concentrations of these variants or longer incubation periods.

### 2.3. Antibacterial Activity and Stability of Modified Molecules in the Sera of Different Animal Species

**Figure 3:**
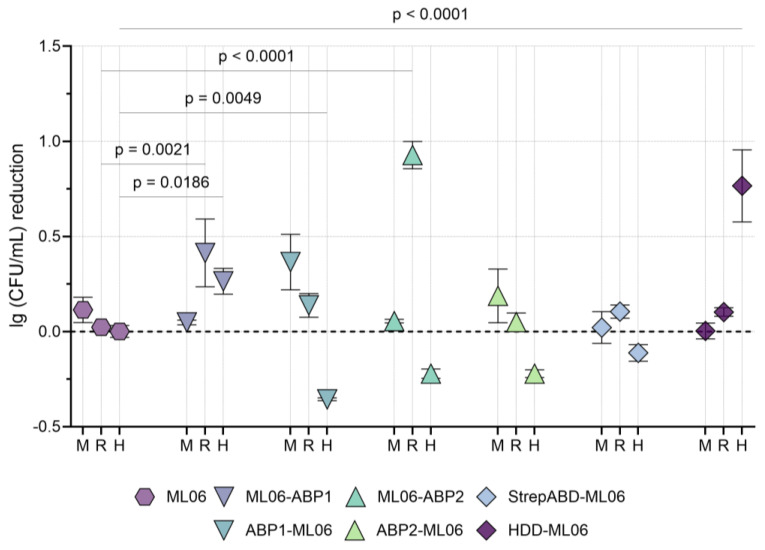
Antibacterial activity of the enzymes in 500 µg/mL concentration against *A. baumannii* 50-16 (60 min incubation) in mouse, rabbit, and human non-inactivated sera. Data are shown as mean lg (CFU/mL) reduction relative to negative control ± SD (two-way analysis of variance, data are compared to ML06 group). M—mouse, R—rabbit, and H—human serum.

The antibacterial effect of the modified enzymes in pools of non-inactivated blood serum from mice, rabbits, and humans was studied using the model *A. baumannii* strain. In all three serum variants, the non-modified ML06 was found to be inactive, as well as StrepABD-ML06 (Figure 3).

In mouse serum, all differences from ML06 were not statistically significant, with a tendency for proteins with N-terminus modifications to be more active than those with C-terminus ABDs. For rabbit serum, on the contrary, ML06-ABP1 and ML06-ABP2 were active (mean values of 0.41 and 0.93 lg (CFU/mL) reduction, respectively), and significantly superior to unmodified protein. In human serum, many of the proteins tested resulted in the additional growth of bacterial cultures, but ML06-ABP1 and HDD-ML06 showed significant bactericidal activity (0.26 and 0.77 lg (CFU/mL) reduction, respectively).

Based on the in vitro antibacterial activity data, including activity in blood sera from different animal species, the molecules ML06-ABP1, ML06-ABP2, and HDD-ML06 were selected for further studies. This allows us to investigate three different types of modifications: two different albumin-binding peptides at the C-terminus of the endolysin and a homodimerization domain at the N-terminus. StrepABD-ML06 was not considered for further study due to poor stability in solution and complete inhibition in the presence of blood serum.

To assess the degradation of the selected molecules under the action of blood proteases, we provided Western blot analysis of modified endolysins incubated in pools of non-inactivated blood sera from mice, rabbits, and humans over 2 h. ML06 was shown to be almost completely degraded within 2 h in mice sera (Figure S2). A truncated protein, presumably LysAp22 without the SMAP peptide, was detected. ML06-ABP1 and ML06-ABP2 showed two-step degradation dynamics (Figures S3 and S4), primarily to ML06 and further to LysAp22 endolysin. Notably, the parent LysAp22 enzyme did not degrade to lower-molecular-weight derivatives during the incubation period studied. HDD-ML06 did not undergo significant degradation—we only detected truncated versions of the co-expressed admixture ML06 and traces of LysAp22, most likely related to the degradation of ML06 (Figure S5). Similar degradation dynamics was observed for the enzymes in the rabbit and human sera, but in all cases, the proteins were degraded at a slower rate. HDD-ML06 also showed the most promising picture and did not degrade significantly.

The obtained results show at least the partial preservation of the modified enzymes in serum for at least 2 h and a significantly slower degradation of ML06 to the original fragments, which will definitely lead to a loss of activity in the molecule. Thus, these modifications can be considered promising for obtaining antibacterial agents, but in vivo degradation patterns may be much more complex. In this context, a pharmacokinetic study in mice was performed for ML06-ABP1, ML06-ABP2, and HDD-ML06.

### 2.4. Comparison of Pharmacokinetic Properties of ML06 and Its Modifications

A comparative pharmacokinetic study in mice was conducted for four proteins (ML06-ABP1, ML06-ABP2, and HDD-ML06, as well as the parent ML06) via a single intravenous administration of 10 mg/kg of each protein (approximately 200 µg per animal).

**Figure 4:**
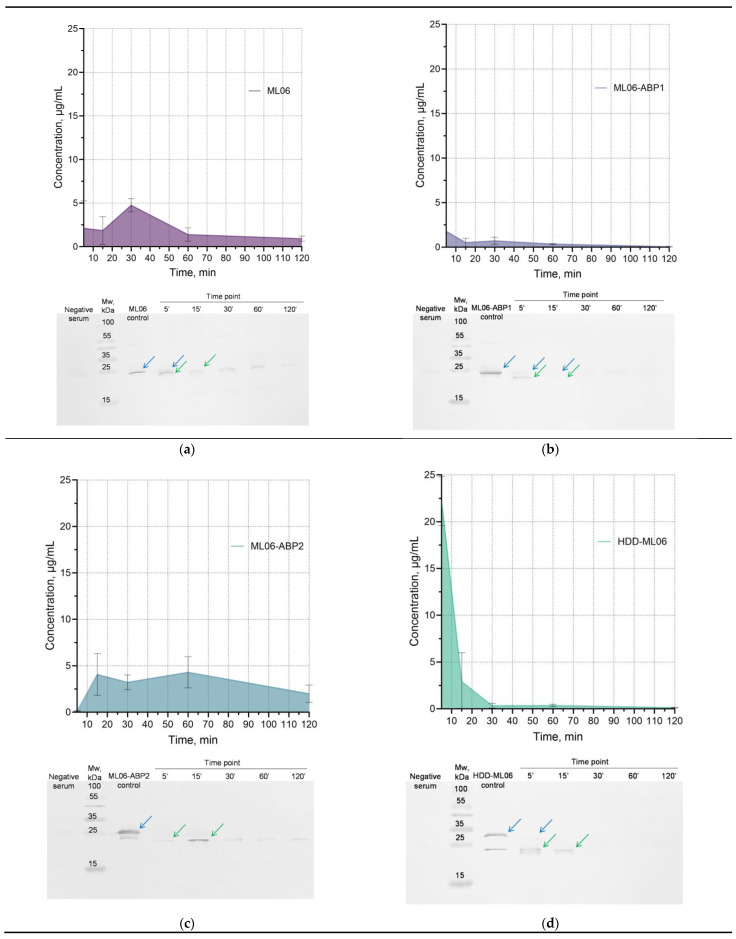
The ELISA-detected endolysins murine serum concentration–time curve after a single intravenous injection of: (**a**) ML06; (**b**) ML06-ABP1; (**c**) ML06-ABP2; and (**d**) HDD-ML06 at the dose of 10 mg/kg. Mean values (n = 4 for a time point) ± SD are present. “Negative serum”—negative mice serum; Mw, kDa—molecular weight marker; and “Control”—positive control, the corresponding enzyme in negative serum. Blue arrows indicate non-degraded enzyme, green arrows indicate major fragments of degradation.

The concentration of parent ML06 in blood serum does not exceed 5 µg/mL (at 30 min time point), and its time–concentration curve has been shown to be rather atypical for bolus intravenous drug administration (Figure 4a). We speculate that the increase in concentration may be due to the degradation of the enzyme in the blood into multiple fragments (Figure S2), resulting in an increase in the amount of detectable epitopes and the total concentration of ELISA-detectable fragments, which then decrease via the standard elimination mechanisms. We have shown using Western blot analysis that ML06 is partially degraded, presumably to the LysAp22 molecule, within the first few minutes in blood, and that the combination of the two proteins is present for 1 h and then becomes almost undetectable.

The ML06-ABP1 modification showed much lower blood concentrations (1.76 µg/mL at 5 min) compared to ML06 (Figure 4b). Western blot and ELISA also showed that only insignificant amounts of the protein were present even 15 min after injection. The ML06-ABP2 enzyme has similar kinetics to ML06 in blood serum, including degradation dynamics (Figure 4c). The protein concentration in the blood increases, remains at a plateau (4.1–4.3 µg/mL) between 15 and 60 min and then gradually decreases to a relatively high concentration (2.0 µg/mL) at 2 h, while the concentration of other enzymes decreases to less than 1.0 µg/mL at the 2 h time point. The time–concentration curve of HDD-ML06 was consistent with a standard two-compartment model (Figure 4d), with a maximum concentration of 22.2 µg/mL, although the enzyme was almost completely degraded to lower-molecular-weight fragments by the 5 min point (as were the other proteins studied) and also almost undetectable by 30 min after injection.

The pharmacokinetic parameters were calculated using non-compartmental analysis (Table 4), which is a universal approach to analyzing data without making assumptions about a specific number of model compartments and allows for general information about the kinetics of a drug in the body to be evaluated [23,24].

We have shown that the half-lives of ML06-ABP2 and HDD-ML06 were increased by 1.7 and 1.6 times, respectively, compared to the ML06 half-life of 45.34 min, but that the ML06-ABP1 t_1/2_ was decreased by 1.6 times. Meanwhile, MRT changed in a different way, increasing in the case of ML06-ABP2 (by 1.2 times compared to ML06) and decreasing in the case of ML06-ABP1 and HDD-ML06 (by 1.3 and 3.4 times, respectively), as they were eliminated much faster than the others, which was confirmed by the total clearance data. The AUC values significantly decreased by 4.6-fold for ML06-ABP1, increased by 1.6-fold for ML06-ABP2, and did not change for HDD-ML06.

Meanwhile, ML06-ABP2 modification did not increase the detected serum concentrations (up to 4.3 µg/mL), but increased the half-life and MRT by 1.6 and 1.2 times (to 1.29 h and 0.98 h, respectively). The highest detected serum concentrations were shown for HDD-ML06 (22.2 µg/mL), which is 4.6 times higher than the parent ML06, and the half-life was also increased by 1.6 times (to 1.22 h), but the MRT value was the lowest (0.23 h), as this hybrid molecule was cleared much faster than others.

Taking into account the dynamics of organ distribution, the highest enzyme concentrations were found in the kidneys and liver, as these are the main organs for excretion and degradation (Figure S6). We can see that ML06-ABP1 is detected at very low levels in the kidney, liver, and spleen (less than 1 µg/mL). ML06-ABP2 was present in the kidneys (up to 1.3 µg/mL), liver (up to 1.6 µg/mL), and spleen (up to 1.4 µg/mL) at relatively high concentrations, with a peak at 15 min post injection; the urea concentration peaked at 60 min and then decreased. The HDD-ML06 organ dynamics reflected the serum kinetics; maximum values of 2.0, 4.1 and 1.9 µg/mL were detected at the 5 min point for the kidneys, liver, and spleen, respectively, and then gradually decreased. Interestingly, the parent enzyme ML06 was mainly detected in the liver (up to 1.2 µg/mL) and spleen (up to 2.2 µg/mL), and almost not in the kidneys (up to 0.3 µg/mL), but its concentration in urea increased over time. In total, the modified enzymes’ organ distribution was similar to the serum time–concentration curve dynamics, so we can propose that the distribution was vascularization-dependent.

## 3. Discussion

The engineering of bacteriolytic enzymes has enormous potential for a wide range of clinical applications. To date, several generations of lytic enzymes have been described, each solving a different problem [10]. Native, non-modified enzymes represent the first generation, which have several problems to be improved. For example, Gram-negative bacteria have a negatively charged outer membrane that inhibits endolysin activity from without. Increasing bactericidal activity by fusing enzymes to permeabilizing peptides is classified as the second generation. The third generation addresses clinical application issues such as improving the pharmacokinetic or pharmacodynamic properties to achieve long-lasting and pronounced antibacterial effects in humans. However, the enzymes that are currently used in clinical trials contain only unmodified molecules (first generation) [25,26,27,28] and can lack clinical relevance, as some studies have shown [29]. It is now clear that the structure of bacteriolytic enzymes must be modified and their properties optimized in order to obtain clinically applicable drugs.

One of the challenging but highly important application areas is the treatment of systemic infections, but lytic enzyme-based drugs, which have been successful as novel alternative therapeutics based on the in vitro and in vivo data [30,31], have limitations partly related to the rate of elimination from the systemic bloodstream.

Several attempts have been made to improve the pharmacokinetic properties of peptidoglycan hydrolases, but not all of them can be considered successful, as not every modification allowed the enzymes to maintain activity [10]. For example, the antistaphylococcal endolysin LysK was fused to different types of albumin-binding domains and its half-life was increased (23 h vs. 34 h), while the antibacterial activity remained [14]. M23 and CH-GH15 antistaphylococcal enzyme half-lives in mice improved by 1.5–2-fold, as well as the in vivo antibacterial activity of hybrid molecules in murine *S. aureus*-induced bacteremia [32]. Also, a well-known antistaphylococcal enzyme, lysostaphin, was modified with an ABD from streptococcal protein G, its half-life in rats was increased fivefold (1.5 h vs. 7.4 h), and its antibacterial activity in serum was also prolonged [15].

A homodimerization strategy of fusing lysostaphin with an anti-parallel α-helical dimerization domain was applied [16]. The hybrid molecule showed a twofold increase in half-life in rats (1.5 h vs. 3.1 h), but the staphylococcal activity in vitro was significantly reduced. On the contrary, the dimerization of the antipneumococcal lysin Cpl-1 by introducing specific cysteine residues resulted in a twofold increase in antibacterial activity and a tenfold decrease in the clearance rate in mice [17].

In the article, the endolysin ML06, modified by the antimicrobial peptide, was studied. We did not include native endolysin without peptides because it was shown to be completely inhibited by isotonic salt concentrations [33]. In selecting the target modifications, we relied on the experience gained in the genetic engineering of lytic enzymes active against Gram-positive bacteria (lysostaphin, Cpl-1, LysK, and others), since these have led to inspiring results. It was decided to introduce albumin-binding sequences from 11 to 45 aa into the structure, as well as a homodimerization domain of 45 aa, because they are small in size and should not significantly affect the biochemical and antibacterial properties of the enzymes. In addition, they can be easily expressed in the heterologous expression system of *E. coli*, ensuring the ease of production and the absence of post-translational modifications that might occur during expression in eukaryotic cells.

The study identified several important aspects that influence the properties of the ML06 enzyme. We showed that almost all modifications reduced the total protein yield, except for StrepABD-ML06, which had the highest yield but low stability in solution. A particularly pronounced decrease in biomass was observed for C-terminal modifications, which may be related to protein toxicity. At present, it is rather difficult to assume a specific mechanism of influence of the modifications, but as shown in a number of studies, the typical structures located at the C-terminus of lysozyme-like muramidases (including GH24), in general, play an important role in the activity of enzymes similar to ML06 [34]. Such structures are currently being considered as potential antimicrobial peptides that allow the enzymes to penetrate bacterial cell membranes and influence the toxicity of the proteins to the producers. The structure of ML06 includes the predicted intrinsically disordered region (IDR) at the C-terminus from 164 to 195 aa (32 aa long). These regions are capable of non-specific binding to various molecules and cellular structures and affect the correct folding of the protein [35]. Additional peptides or domains at the C-terminus of ML06 lead to an elongation of the predicted IDR, while N-terminal modifications do not affect the IDR. IDRs are known to influence protein misfolding and aggregation due to their structural flexibility and lack of tertiary structure [36], leading to the formation of inclusion bodies. We can also speculate that the toxicity to the *E. coli* host may be conditioned by the non-specific binding of the produced enzyme to some essential bacterial molecules; however, this issue needs to be further clarified.

C-terminal modifications were also faster acting, and did not have a pronounced lag phase. The introduction of higher-molecular-weight modifications, such as domains of 45 aa that were introduced via flexible linkers, slows down the activity by approximately 2 times compared to modifications of 11–12 aa, but does not critically decrease the activity in the spectrum of action studies. We have shown that it is possible to retain the initial enzyme activity spectrum and, for some modifications, to improve antibacterial properties. In contrast to others, modification with the albumin-binding peptide ABP1, especially at the C-terminus, significantly increased the activity of the molecules. We can speculate that this is related to the high content of hydrophobic amino acids in ABP1, which may contribute to the amphipathic antimicrobial peptide nature and improve membrane permeation. This can be justified using the well-known Kyte–Doolittle hydropathy scale for the modification sequences [37]. Many more amino acids have a positive score for ABP1 than for ABP2, indicating a significantly higher hydrophobicity (Figure S7). ABP2, StrepABD, and HDD modifications did not affect the spectra as much, and slightly reduced or did not affect the activity against bacterial species. To sum up, no modification had a critical negative effect on the activity of the enzymes.

Prior to in vivo studies, we evaluated the enzyme activity and stability in non-inactivated sera. Only three modifications, ML06-ABP1, ML06-ABP2, and HDD-ML06, were active in sera obtained from different species, while the parent ML06 was completely inactive in serum. Thus, such engineering strategies of Gram-negative bacteria-targeting bacteriolytic enzymes could be applicable for enzybiotics. As was confirmed via Western blot analysis, all enzymes regardless of the modification type appear to degrade stepwise first to ML06 and then hydrolyze via SMAP peptide removal to LysAp22, and the native enzyme is very stable during 2 h incubation. The stability dynamics in human sera were the most promising, as the modified enzymes degraded much more slowly or did not degrade at all. Interestingly, even the ML06 enzyme was more stable in mouse serum than LysECD7-SMAP, which degraded immediately after serum addition in our previous research [38].

The detected ML06 concentration after the single intravenous administration of 10 mg/kg in mice did not exceed 4.8 µg/mL; its half-life was 45.3 min (0.76 h) and MRT was 47.3 min (0.79 h), which is relatively similar to other native or second-generation peptidoglycan hydrolases. For example, non-modified lysostaphin showed a half-life of less than 1 h and a serum concentration of 1 µg/mL at the 1 h time point following the injection of a 40 mg/kg dose in mice [12]. The modified enzyme LysECD7-SMAP at a dose of 12.5 mg/kg showed a half-life of 38.8 min and an MRT of 36.3 min in mice ([38], which is comparable to our data).

As expected, the protein modifications had different effects on the pharmacokinetics of endolysin. Apparently, ML06-ABP1 modification resulted in rapid elimination and did not improve the systemic pharmacokinetics, despite its promising in vitro antibacterial properties. Since Western blot analysis has shown that ML06-ABP1 is quite stable in mouse sera and does not degrade so rapidly in vitro, we can propose that such a dynamic may be related to the instability of the enzyme at high concentrations, which may be due to the introduced modification, or to its potential immunotoxicity, which promotes the faster recognition and degradation of the protein via immune cells in vivo. To clarify this phenomenon, this aspect should be investigated further.

However, an improvement in the pharmacokinetic parameters was observed for ML06-ABP2, with stable levels in the murine blood, and for HDD-ML06, which allowed high concentrations (4.6 times higher than the parent ML06) to be reached in the blood sera in the first few minutes after injection. Thus, we achieved a similar increase in half-life to that of the conjugated Gram-negative antibacterial enzyme V12C-C16 [13], where t_1/2_ increased from 0.4 to 1.5 h.

While the direct genetic modification of bacteriolytic enzymes is not the only approach for the optimization of their properties, it is one of the most widely used. In this context, a comprehensive study provided for ML06 muramidase allowed us to identify a number of features of enzyme engineering. To sum up, C-terminal modifications of lysozyme-like muramidases significantly affect the production of such enzymes, confirming their importance in the structure of lysins. This should be taken into account by researchers when designing enzymes, giving preference to the shorter modifications in the case of small globular endolysins.

Another important observation is the difference in the stability of endolysins in the sera of different animal species, including humans. Taking into account the comparative dynamics of enzyme degradation in the sera of other animals, it is expected that in humans, the pharmacokinetic parameters will be better, allowing the enzymes to circulate long enough to achieve the desired antimicrobial effects. As the rate of metabolism and excretion in mice is high and may not allow for a full assessment of the effects from the introduced modifications, pharmacokinetic data from other species will be of great value in the future.

Our study indicates that the pharmacokinetics of bacteriolytic enzymes that target Gram-negative bacteria can be managed without compromising their antibacterial properties via the genetic engineering of protein molecules. This approach allowed us to obtain two prospective candidates for the further development of effective compositions for the control and treatment of systemic infections caused by antibiotic-resistant Gram-negative pathogens, which would be studied further.

## 4. Materials and Methods

### 4.1. Production and Purification of the Enzymes

ML06 second-generation endolysin consists of the lysozyme-like enzyme sequence LysAp22 (NCBI AN: CCH57765.1), genetically modified with the SMAP-29 (1–17, K2,7,13) peptide at the C-terminus of the protein. The ML06 nucleotide sequence was synthesized in the pAL-TA vector (Evrogen, Moscow, Russia) and introduced into the pET-42b (+) expression vector. All additional modifications were performed using oligonucleotide primers (Table S1, GenTerra, Moscow, Russia). The PCR fragments of the ML06 nucleotide sequence, the modification, and the vector were fused using the Gibson method. All constructs were checked via Sanger sequencing.

The expression vectors of the proteins were introduced into competent cells of *E. coli* Rosetta (Novagen, Darmstadt, Germany) using a standard heat shock transformation protocol. The *E. coli* cells were grown in 2×YT medium with the addition of selective antibiotics (kanamycin, chloramphenicol) at 37 °C, 250 rpm to the exponential phase (OD_600_ = 0.5–0.6), and induced with 1 mM isopropyl β-d-1-thiogalactopyranoside for 4 h. The cells were then harvested via centrifugation (3000× *g*, 4 °C, 15 min).

The harvested cells were resuspended in 1 M phosphate-buffered saline (PBS, pH = 7.4), with the addition of 1 mM phenylmethylsulfonyl fluoride (PMSF) and 0.5 mM EDTA. The suspension was disrupted by sonication (42 Hz, 4′ × 4′ for 45 min). The lysate was then harvested via centrifugation (30 min, 10,000× *g*, 4 °C) and the supernatant or cell debris with inclusion bodies was collected.

For the ML06, ML06-ABP2, and ABP2-ML06 enzymes, two-stage chromatographic purification was performed on the XK 16-600 column (GE Healthcare, Chicago, IL, USA) using ion-exchange SP-Sepharose resin (GE Healthcare, Chicago, IL, USA) and the gel-exclusion Superdex 75pg resin (GE Healthcare, United States). Proteins were eluted with PBS (37 mM NaCl, 2.7 mM KCl, and 10 mM phosphate buffer, pH = 7.3–7.5, VWR, Radnor, PA, state, United States).

For the ML06-ABP1, ABP1-ML06, ML06-StrepABD, StrepABD-ML06, ML06-HDD, and HDD-ML06 enzymes, the inclusion bodies were washed in PBS with 0.1% Triton addition and then dissolved in 8 M urea, 50 mM NaCl, and 20 mM Tris-HCl, pH = 7.9. Chromatographic purification was then carried out using ion-exchange SP-Sepharose resin and gel-exclusion Superdex 75pg resin. The proteins were eluted with 8M urea, 1M NaCl, and 20 mM Tris-HCl, pH = 7.9 and refolded into PBS via the dialysis method.

The final concentrations were determined via OD_280_ measurement (Implen NanoPhometer, IMPLEN, München, Germany) and calculated with the predicted extinction coefficients (0.847 (mg/mL)^−1^cm^−1^ for ML06; 1.275 (mg/mL)^−1^cm^−1^ for ABP1 modifications; 0.796 (mg/mL)^−1^cm^−1^ for ABP2 modifications; 0.803 (mg/mL)^−1^cm^−1^ for StrepABD modifications; and 0.858 (mg/mL)^−1^cm^−1^ for HDD modifications). The protein purity was determined via 16% sodium dodecyl sulphate-polyacrylamide electrophoresis (SDS-PAGE).

### 4.2. Antibacterial Activity Assay

*A. baumannii* 50-16 (clinical isolate from patient’s sputum) bacterial culture grown at 37 °C, 250 rpm for 16–20 h was used as stationary-phase bacterial culture or diluted in Mueller–Hinton broth (MHB, HiMedia Laboratories Pvt. Ltd., Maharashtra, India) and grown to the OD_600_ = 0.5–0.7 (exponential growth phase). The culture was harvested (10 min, 6000× *g*, RT), resuspended in PBS pH = 7.4 to a turbidity of 0.5 McFarland turbidity standard, and diluted 100 times in PBS pH = 7.4 (to ~ 10^6^ CFU/mL) [39].

Subsequently, 100 μL of enzymes or PBS pH = 7.4 (negative control) and 100 μL of bacterial suspensions were mixed in 96-well plates, incubated for 30 min (200 rpm, 37 °C), diluted tenfold in PBS and plated on Mueller–Hinton agar (MHA), followed by incubation at 37 °C for 16–18 h. The CFUs were counted and lg (CFU/mL) reduction values were estimated. All experiments were performed in triplicate.

Protein concentrations of 0.1, 1, 5, 10, and 100 μg/mL were used in the dose–response study against exponential and stationary-phase bacteria. The time–kill assay was performed at a 0.5 μg/mL or 1 μg/mL (for StrepABD-ML06 and HDD-ML06) protein concentration at 5, 10, 30, and 60 min of incubation.

The spectrum study was performed at a concentration of 100 μg/mL on bacterial strains selected from collection strains and clinical isolates (collection of the Gamaleya National Center for Epidemiology and Microbiology, Table S2) of *A. baumannii* (n = 5), *P. aeruginosa* (n = 5), *E. coli* (n = 5), and *K. pneumoniae* (n = 5) at a 60 min incubation time.

The antibacterial activity of the enzymes (500 µg/mL final concentration) in pools of mouse, rabbit, or human non-inactivated sera was evaluated against *A. baumannii* 50-16 at a 60 min incubation time.

The mean values ± SD were calculated. As the data follow a normal distribution (Shapiro–Wilk test), two-way analysis of variance was used to compare the modified enzymes to the ML06 group (Dunnett’s multiple comparisons test).

### 4.3. Pharmacokinetic Study

The animal procedures were approved by the Ethics Committee of the N.F. Gamaleya National Research Center for Epidemiology and Microbiology (protocol number 64, 10 October 2023) and were performed in accordance with the relevant guidelines for the care and use of laboratory animals.

The study used 80 male BALB/c mice (20–22 g, 6–8 weeks, Stolbovaya Farm, Russia). The animals were acclimatized for 2 weeks prior to the experiment and randomly divided and housed into experimental groups: ML06 pharmacokinetics (n = 20), ML06-ABP1 pharmacokinetics (n = 20), ML06-ABP2 pharmacokinetics (n = 20), and HDD-ML06 pharmacokinetics (n = 20), where individual weights differed by more than 10%.

Solutions of ML06, ML06-ABP2, and HDD-ML06 (100 µL) were injected into the tail veins of mice (n = 4 for each time point) at a dose of 10 mg/kg. After single injection at 5, 15, 30, 60, and 120 min time points [38], the animals were anesthetized via the intramuscular injection of a zoletil-xylazine mixture (45 mg/kg and 7.5 mg/kg). Blood samples were collected via heart puncture into clot activator tubes and centrifuged (10 min, 3000× *g*, RT) to obtain serum. After blood sample collection, animals were sacrificed via cervical dislocation, organs (liver, spleen, kidneys, and muscles), excised, and thoroughly washed in sterile PBS solution (pH = 7.4); urine samples were collected. All samples were frozen and stored at −80 °C prior to further processing. Organs were then weighed and homogenized in PBS (pH = 7.4) with the addition of 1 M NaCl, 0.5 mM EDTA, and 1 mM PMSF using a TissueLyser II, 30 Hz, 4 min (Qiagen, Germany); homogenates were centrifuged (10 min, 10,000× *g*, 4 °C); and supernatants were collected. The concentrations of modified enzymes were measured by the in-house enzyme-linked immunosorbent assay (ELISA) using ML06-specific rabbit antibodies, using standard curves for each protein at concentrations ranging from 2.5 to 40 ng/mL.

The pharmacokinetic parameters were estimated using standard non-compartmental equations [23,24] using Microsoft Excel and GraphPad Prism 10 software. The mean values ± SD were calculated. As the data follow a normal distribution (Shapiro–Wilk test), two-way analysis of variance was used to compare the modified enzymes to the ML06 group (Dunnett’s multiple comparisons test).

### 4.4. Enzyme-Linked Immunosorbent Assay

Rabbits were immunized with ML06 (0.25 mg per one immunization) with Freund adjuvant 6 times at 10–14-day intervals to obtain hyperimmune serum containing specific polyclonal antibodies. Specific antibodies were purified from hyperimmune serum using affinity chromatography with amynoethyl sepharose sorbent (NHS-Sepharose, GE Healthcare, Düsseldorf, Germany) conjugated to ML06. The antibody concentration was measured at OD_280_, and the specific activity was confirmed using indirect ELISA.

Wells of high sorption 96-well ELISA plates (Servicebio, Wuhan, China) were coated overnight with 1 μg of purified ML06-specific polyclonal antibody in carbonate-bicarbonate buffer pH = 9.3–9.6 at 4 °C. The next day, the plates were blocked overnight with S002X blocking buffer (Xema, Moscow, Russia) with the addition of 5% sucrose and 0.5% sorbitol. The liquid was then removed and the plates dried for 48 h at room temperature, then placed in plastic bags and stored at 4 °C prior to use.

In the experiment, serum, homogenates, and urine samples were diluted in PBS-NTBP (PBS pH = 7.4, 0.5 M NaCl, 1% BSA, 0.1% Tween 20, 1% ProClin™ 300 (Sigma-Aldrich, St. Louis, MO, USA)) in a ratio of 1:100 and 1:1000 for serum, 1:100 for homogenates and 1:20 for urine, added to wells (100 μL), and incubated for 1 h at 37 °C and 600 rpm. The wells were washed three times with S008 buffer solution (Xema, Moscow, Russia) to remove unbound antigens, and 100 μL of HRP-conjugated-ML06 rabbit IgG was added at a dilution of 1:10,000 and incubated for 1 h at 37 °C and 600 rpm, and then washed six times with S008.

To visualize the reaction, tetramethylenebenzidine (TMB) (R055, Xema, Moscow, Russia) was added and incubated for 10 min at room temperature in the dark. The reaction was stopped with 10% HCl solution and the OD_450_ was measured (Multiscan FC, Thermo Scientific, Waltham, MA, USA).

The following parameters were estimated: ML06 analytical sensitivity was 0.086 ng/mL for serum and 0.226 ng/mL for homogenates; ML06-ABP1 analytical sensitivity was 0.178 ng/mL for serum and 0.281 ng/mL for homogenates; ML06-ABP2 analytical sensitivity was 0.301 ng/mL for serum and 0.472 ng/mL for homogenates; and HDD-ML06 analytical sensitivity was 0.112 ng/mL for serum and 0.252 ng/mL for homogenates.

The lower limits of quantity (LLOQ) were 0.462 ng/mL and 0.608 ng/mL for ML06 in serum and homogenates, respectively; 0.558 ng/mL and 0.664 ng/mL for ML06-ABP1; 0.859 ng/mL and 0.994 ng/mL for ML06-ABP2; and 0.636 ng/mL and 0.786 ng/mL for HDD-ML06.

The linear range of ML06 detection was 0.46–40 ng/mL, which, considering minimal dilution factors, is equal to 46–4000 ng/mL in samples. The linear range of ML06-ABP1 detection was 0.56–20 ng/mL, which is equal to 56–2000 ng/mL in samples; the linear range of ML06-ABP2 detection was 0.86–40 ng/mL (86–4000 ng/mL in samples) and 0.64–40 ng/mL (64–4000 ng/mL in samples) for HDD-ML06. Samples with higher concentrations were diluted.

The within-run precision for low- (2.5 ng/mL) and medium (10 ng/mL)-concentration samples was 96.16% and 96.61% for ML06, respectively; 98.34% and 93.2% for ML06-ABP1; 95.53% and 96.4% for ML06-ABP2; and 93.18% and 93.00% for HDD-ML06.

The selectivity of the method was confirmed by measuring the negative samples of 10 animals in the placebo group.

### 4.5. Western Blot Analysis

The integrity of endolysin in mouse sera during the pharmacokinetic study was verified via Western blot analysis [38]. Initially, 16% SDS-PAGE was performed on 10 μg samples. The gel was then placed in Towbin buffer solution (25 mM Tris-HCl pH = 7.5, 192 mM glycine, 20% ethanol) for 10 min, RT, 250 rpm. The proteins were then transferred to the nitrocellulose membrane (Bio-Rad, Hercules, CA, USA) using the Trans-Blot Turbo System (Bio-Rad, Hercules, CA, USA). Nitrocellulose membranes were blocked with TBST with 1% BSA (TBST-BSA, 20 mM Tris pH = 7.5, 150 mM NaCl, 0.1% Tween-20, 1% BSA) blocking buffer for 10 min in a SNAP i.d. 2.0 Protein Detection System (Merck, Millipore, Darmstadt, Germany), and then incubated with anti-ML06-specific antibodies (2 μg/mL) diluted in TBST-BSA buffer solution for 10 min, RT. The blot was washed four times with TBST and the HRP-conjugated anti-rabbit IgG antibodies (HyTest, Moscow, Russia) diluted 1:5000 in TBST-BSA buffer were added to the blot and incubated for 10 min, RT. The membrane was then gently washed five times with TBST buffer solution. To visualize the reaction, TMB was added and incubated for approximately 2 min, RT. The reaction was stopped by washing the membrane with ultrapure water.

For the in vitro stability assay, ML06, ML06-ABP1, ML06-ABP2, and HDD-ML06 were added to non-inactivated negative mouse, rabbit, and human sera to a final amount of 10 μg and incubated at 37 °C for 5, 15, 30, 60, and 120 min. Serum samples without endolysins were used as a negative control. Non-modified LysAp22, ML06, ML06-ABP1, ML06-ABP2, and HDD-ML06 in PBS and in corresponding negative sera were used as molecular weight controls.

## Figures and Tables

**Table 1:** Variants of ML06 endolysin modifications.

The Protein Name	Amino Acid Sequence of Modification	Linker	C- or N-Terminus	Mw, kDa
ML06	-	-	-	22.49
**Albumin-Binding Peptides/Domains**				
ML06-ABP1	DICLPRWGCLW	-	C-	23.97
ABP1-ML06			N-	
ML06-ABP2	LPHSHRAHSLPP	-	C-	23.95
ABP2-ML06			N-	
ML06-StrepABD	AEAKVLANRELDKYGVSDYYKNLINNAKTVEGVKALIDEILAALP	Flexible linker GSAGSAAGSGEF	C-	28.52
StrepABD-ML06			N-	
**Dimerization**				
ML06-HDD	MKQLEKELKQLEKELQAIEKQLAQLQWKAQARKKKLAQLKKKLQA	Flexible linker GSAGSAAGSGEF	C-	28.84
HDD-ML06			N-	

**Table 2:** Biotechnological product characteristics of ML06 modifications.

The Protein Name	Biomass Yield, g/L	Solubility	Protein Yield, mg/g
ML06	3.75	SN	3.97
ML06-ABP1	2.4	IB	2.44
ABP1-ML06	3.67	IB	0.79
ML06-ABP2	1.99	SN	1.36
ABP2-ML06	3.55	SN	3.1
ML06-StrepABD	1.77	IB	-
StrepABD-ML06	2.55	IB	10.3
ML06-HDD	2	IB	-
HDD-ML06	4.7	IB	2.37

SN, supernatant (soluble fraction); IB, inclusion bodies (insoluble fraction).

**Table 3:** Calculated ED50 effective doses of ML06 modifications against exponential and stationary phase *A. baumannii* 50-16 bacteria.

The Protein Name	Exponential Phase ED50, µg/mL	Stationary Phase ED50, µg/mL
ML06	0.167	4.262
ML06-ABP1	0.247	3.384
ABP1-ML06	0.394	3.939
ML06-ABP2	0.192	7.124
ABP2-ML06	0.327	4.71
StrepABD-ML06	1.185	13.47
HDD-ML06	1.54	20.46

**Table 4:** Calculated systemic pharmacokinetic parameters of ML06 and modified endolysins.

Parameter	ML06	ML06-ABP1	ML06-ABP2	HDD-ML06
t_1/2_, min	45.34 ± 13.82	29.24 ± 17.18	77.66 ± 55.35	72.98 ± 33.45
MRT, min	47.31 ± 4.81	37.37 ± 4.57	58.58 ± 3.34	14.06 ± 2.44
AUC_0–∞_, min × μg/mL	227.2 ± 35.22	49.2 ± 16.7	370.5 ± 125.2	207.9 ± 79.5
Cl_T_, mL/min	0.90 ± 0.15	1.66 ± 0.54	0.59 ± 0.21	1.06 ± 0.34

AUC_0–∞_, area under the curve; Cl_T_, total clearance; kel, elimination rate constant; MRT, mean residence time; and t_1/2_, half-life.

## Data Availability

The original contributions presented in this study are included in the article/Supplementary Material. Further inquiries can be directed to the corresponding author.

## Supplementary Materials

The following supporting information can be downloaded at: https://www.mdpi.com/article/10.3390/ijms26094376/s1.

## Abbreviations

The following abbreviations are used in this manuscript:

ABP	Albumin-binding peptide
ABD	Albumin-binding domain
HDD	Homodimerization domain
