# Supplementary material for: Modifying Pharmacokinetic Properties of the Gram-Negative Bacteria Targeting Endolysin ML06 Without Affecting Antibacterial Activity
Source: Int J Mol Sci. 2025 May 4;26(9):4376. doi: 10.3390/ijms26094376 (PMC12072273; doi:10.3390/ijms26094376)
Supplement: Supplementary file 1 [file ijms-26-04376-s001.zip › ijms-3561716-supplementary.pdf]

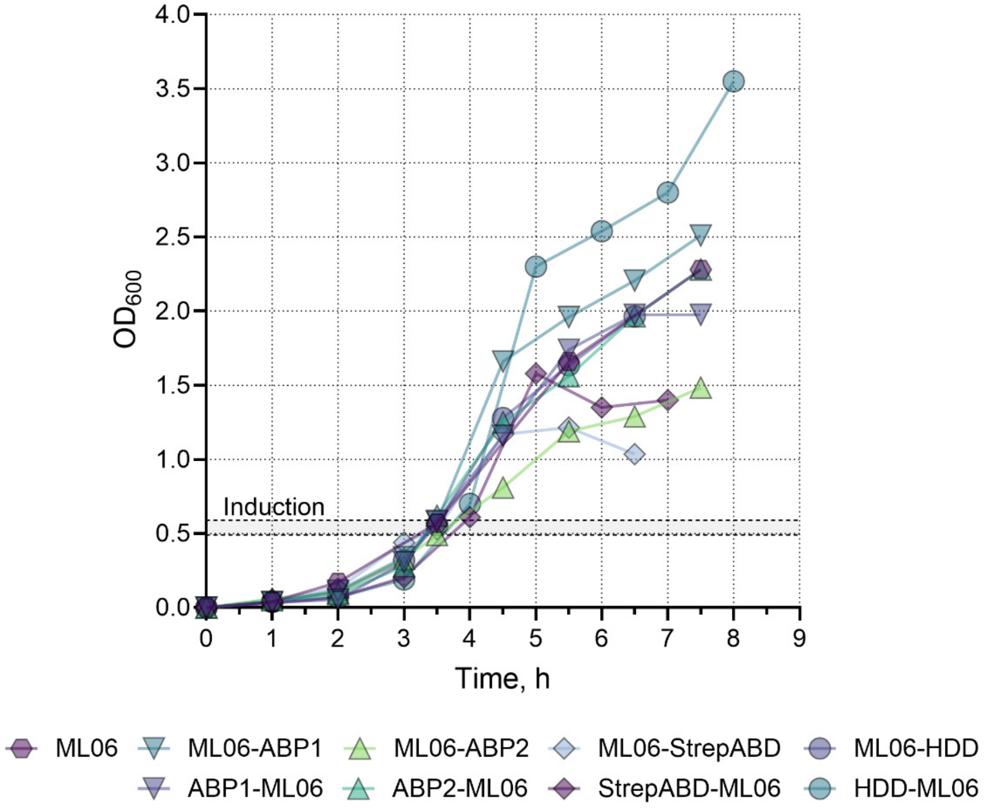

Figure S1. Growth curves of *E. coli* Rosetta expression strains carrying vectors encoding the modified endolysin sequences.

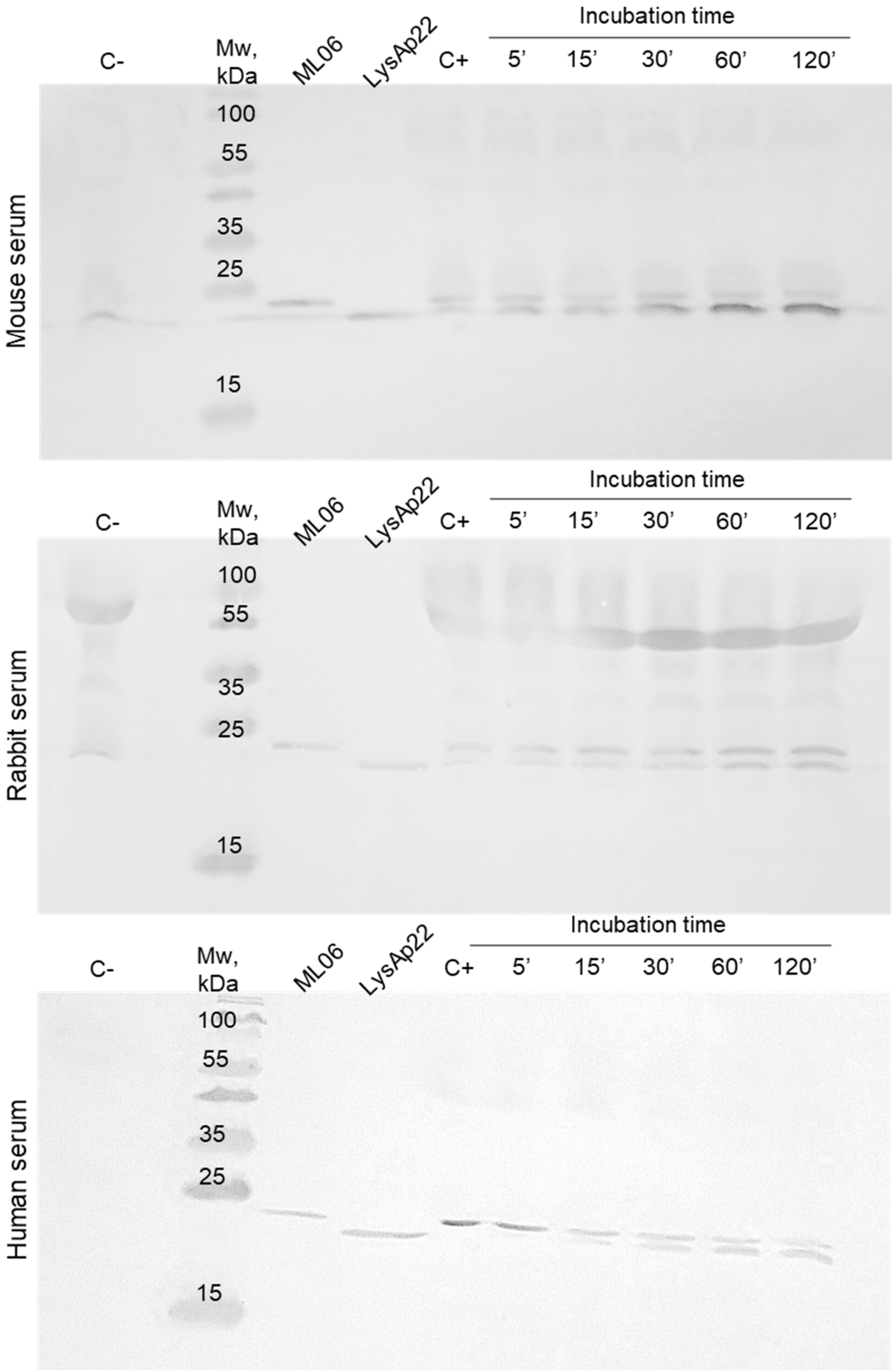

Figure S2. Western blot analysis of ML06 incubated in mouse, rabbit and human serum samples. "C-" - negative serum; Mw, kDa – molecular weight marker; the corresponding enzyme control samples in PBS; "C+" - positive control, the corresponding enzyme in negative serum; the corresponding enzyme incubated in negative serum for 5 min - 2 h period.

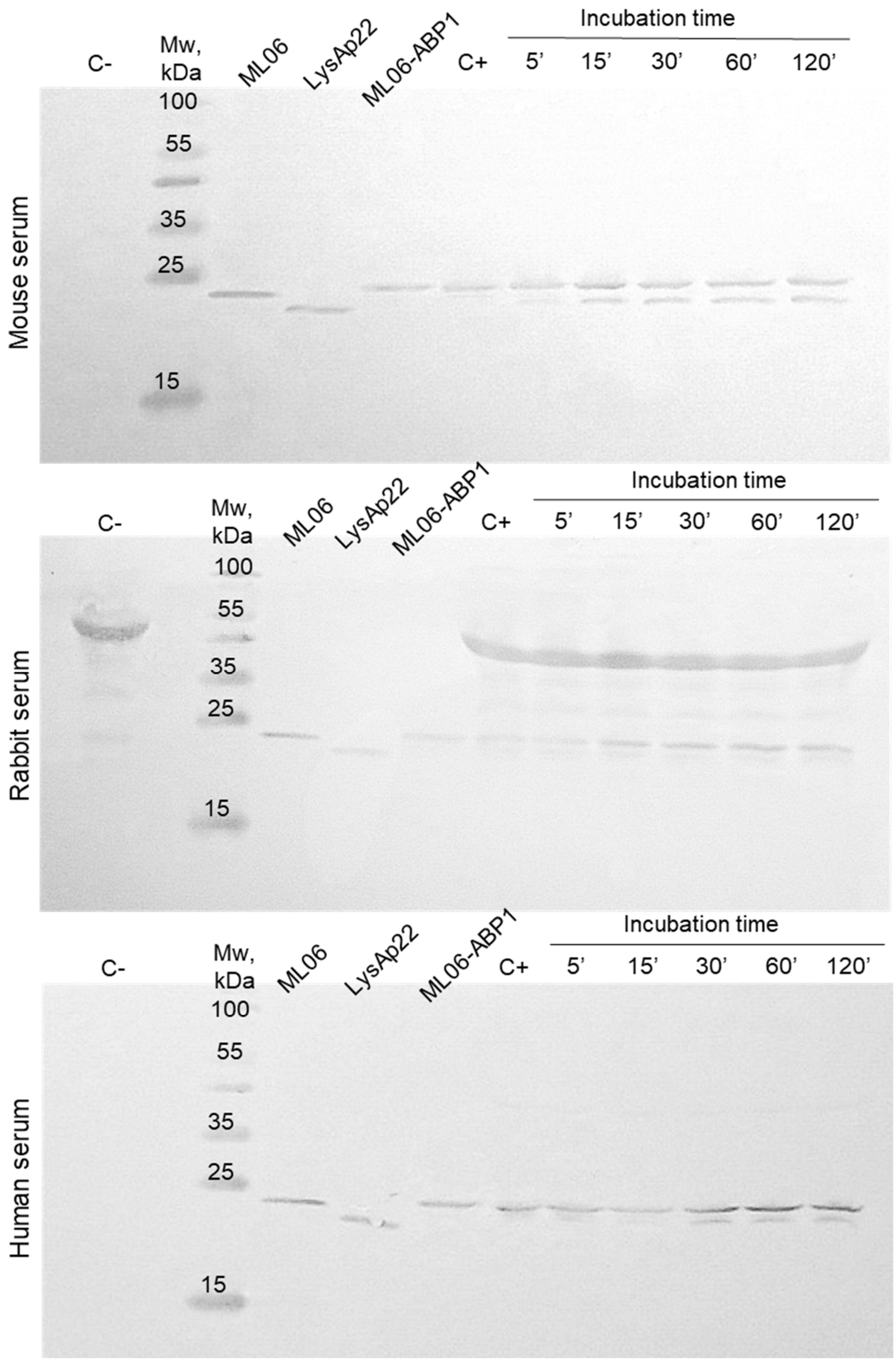

Figure S3. Western blot analysis of ML06-ABP1 incubated in mouse, rabbit and human serum samples. "C-" - negative serum; Mw, kDa – molecular weight marker; the corresponding enzyme control samples in PBS; "C+" - positive control, the corresponding enzyme in negative serum; the corresponding enzyme incubated in negative serum for 5 min - 2 h period.

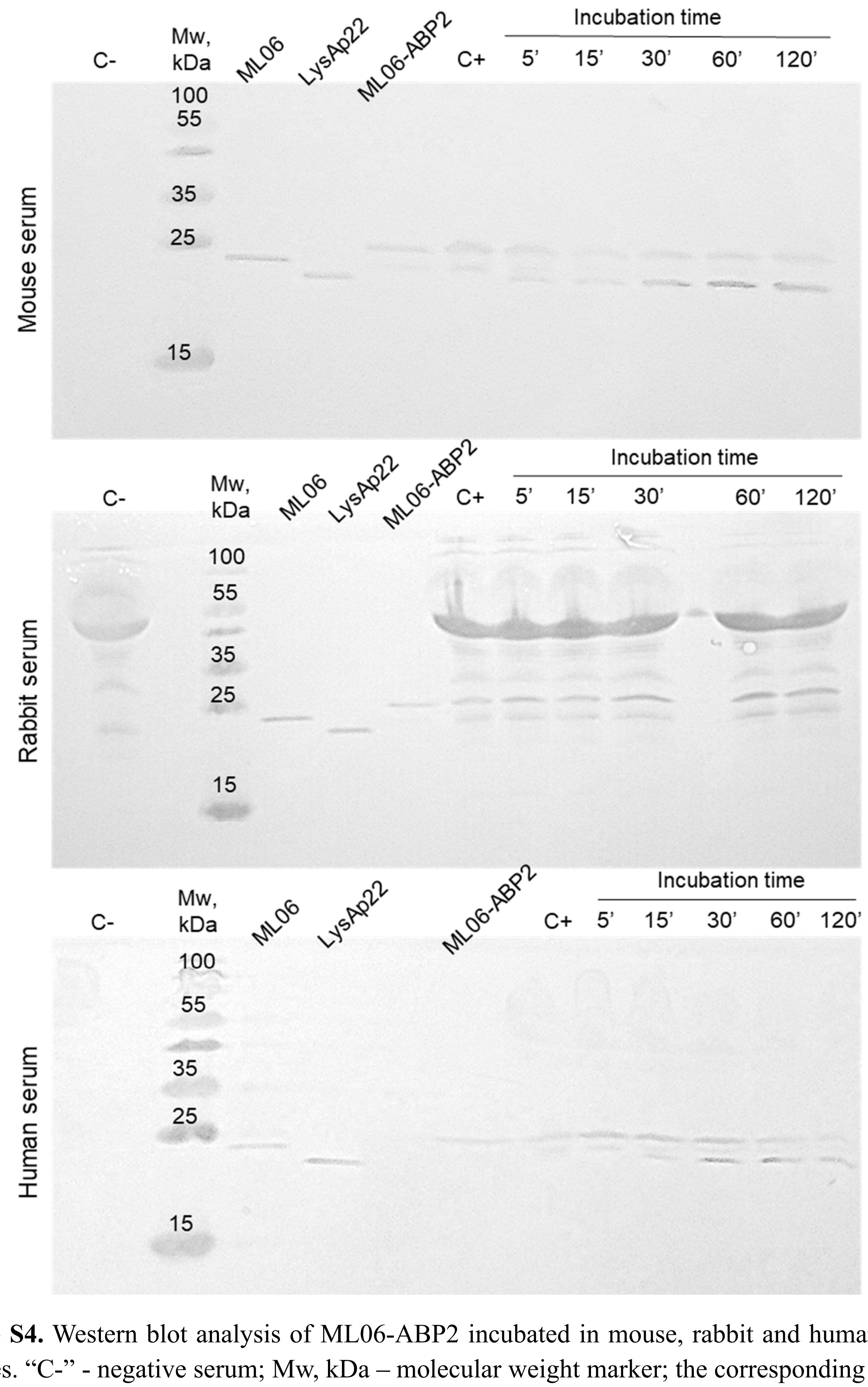

Figure S4. Western blot analysis of ML06-ABP2 incubated in mouse, rabbit and human serum samples. “C-” - negative serum; Mw, kDa – molecular weight marker; the corresponding enzyme control samples in PBS; “C+” - positive control, the corresponding enzyme in negative serum; the corresponding enzyme incubated in negative serum for 5 min - 2 h period.

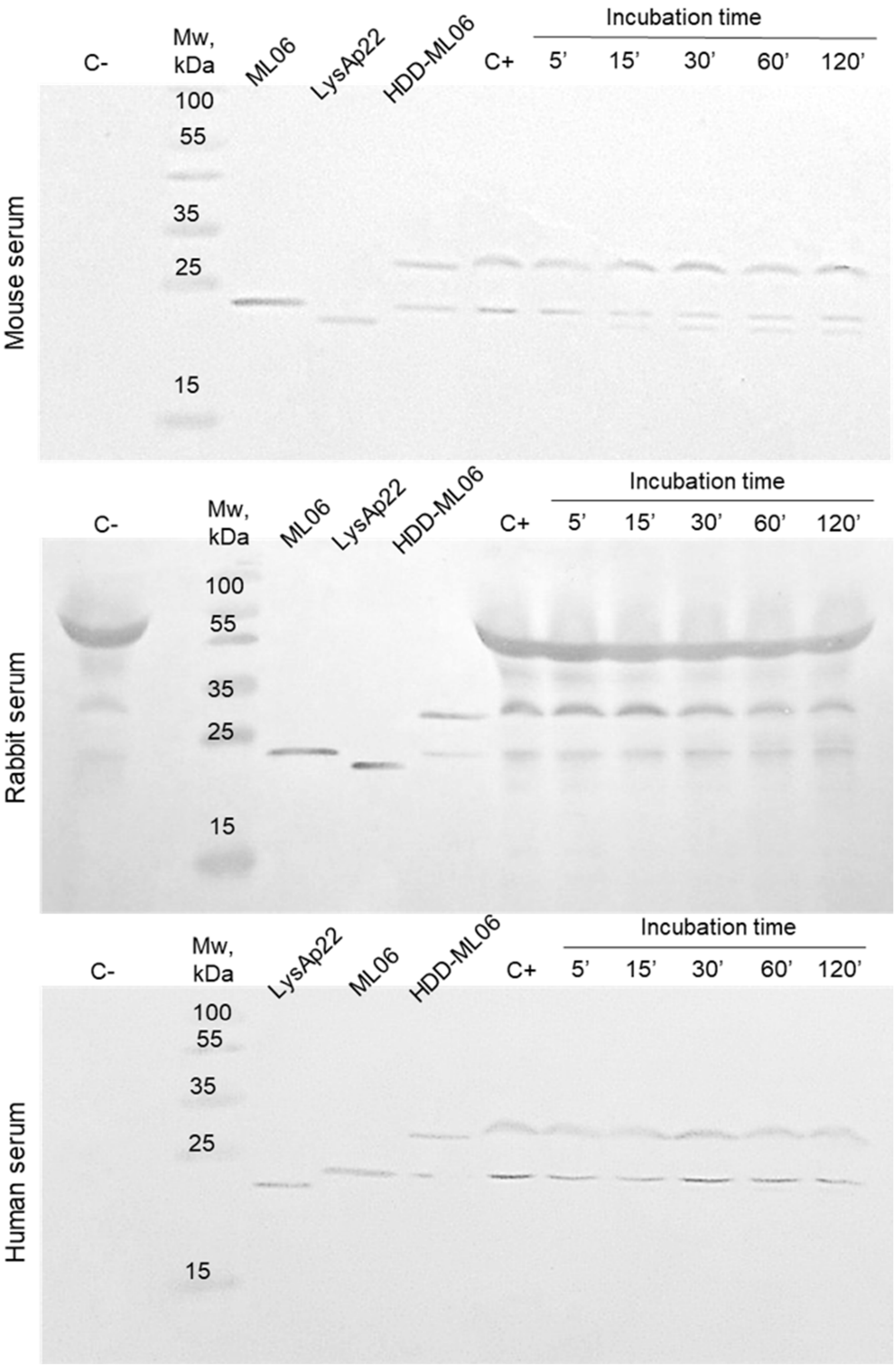

Figure S5. Western blot analysis of HDD-ML06 incubated in mouse, rabbit and human serum samples. "C-" - negative serum; Mw, kDa – molecular weight marker; the corresponding enzyme control samples in PBS; "C+" - positive control, the corresponding enzyme in negative serum; the corresponding enzyme incubated in negative serum for 5 min - 2 h period.

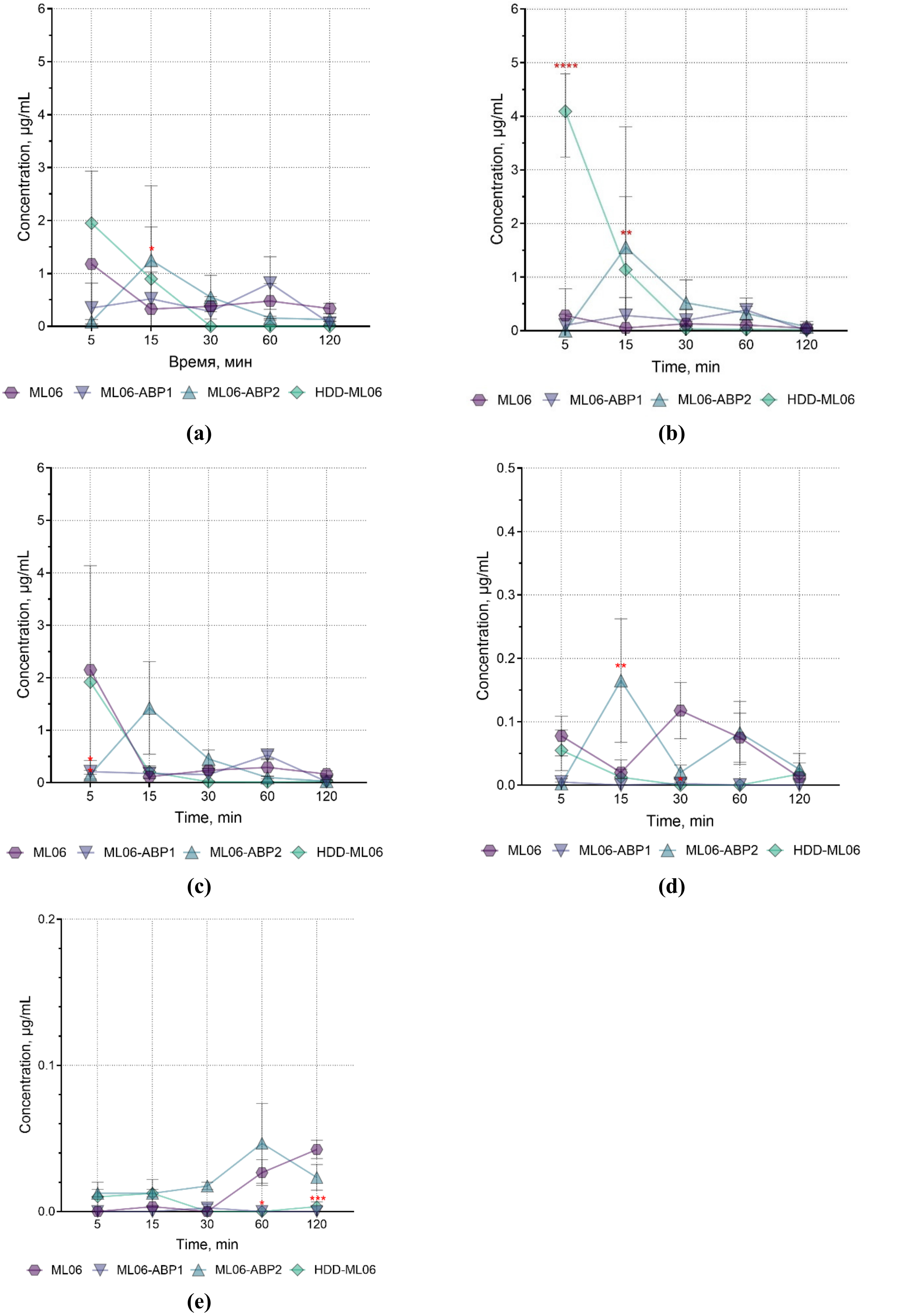

Figure S6. The ELISA-detected endolysins concentration-time curve after a single intravenous injection at the dose of 10 mg/kg in: (a) kidney; (b) liver; (c) spleen; (d) muscles; (e) urea. Mean values (n=4 for a time point) ± SD are present. Significant differences from ML06 group (two-way analysis of variance) are shown; * - $p<0.05$, ** - $p<0.01$, *** - $p<0.001$, **** - $p<0.0001$

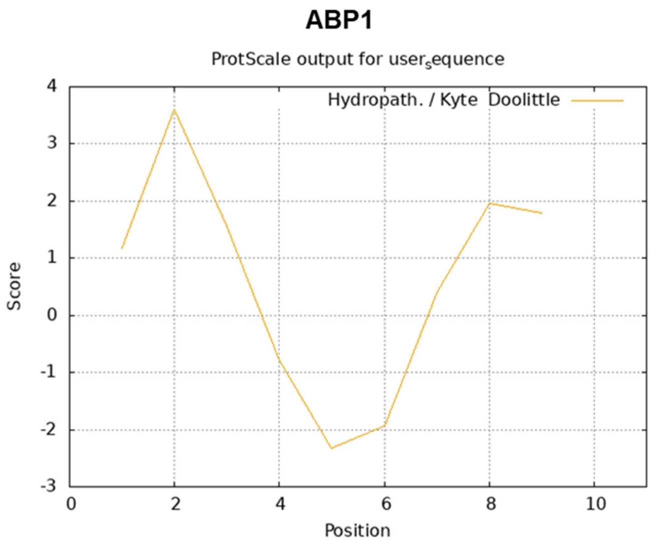

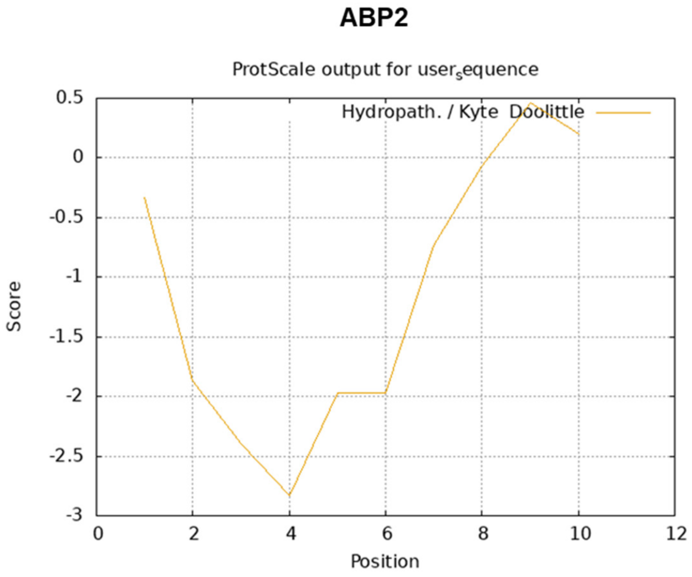

Figure S7. The predicted Kyte-Doolittle hydropathy score for the ABP1 and ABP2 sequences calculated using ProtScale Hydropathicity tool (https://web.expasy.org/protscale/).

Table S1. Primers used in the study.

Enzyme	Primer	Sequence of oligonucleotide primers, 5'–3'
ML06-ABP1	ACPvecF	CGAAAGGAAGCTGAGTTGGCTGCT
	ABP1smapR	ACAACCCCAACGAGGCAGACAAATATCCATATACTTCTTCACCTTATGTGCTATCTTCCTCTTC
	ABP1rbsF	TCTGCCTCGTTGGGGTTGTCTGTGGTAAGGGAATTAGAAGAATGAGTGTGATCGCTAAAC
	ACPR	AGCCAACTCAGCTTCCTTTCGCTAGGCGACCACAGGTTTGCG
ABP1-ML06	ACPvecF	CGAAAGGAAGCTGAGTTGGCTGCT
	ABP1VR	ACAACCCCAACGAGGCAGACAAATATCCATATGTATATCTCCTTCTTAAAGTTAAACAAAATTATTTC
	rbsAmCPF	GGGAATTAGAAGAATGAGTGTGATCGCTAAACAAATGACCTAC
	ACPR	AGCCAACTCAGCTTCCTTTCGCTAGGCGACCACAGGTTTGCG
	ABP1AP22F	TCTGCCTCGTTGGGGTTGTCTGTGGATGAGTATTAAGAATTTCTTTGATGCTGCGC

	rbssmapR	CGATCACACTCATTCTTCTAATTCCCTTAATACTTCTTCACCTTATGTGCTATCTTCCTCTTC
ML06-ABP2	ACPvecF	CGAAAGGAAGCTGAGTTGGCTGCT
	ABP2smapR	AGACTATGTGCACGATGACTGTGAGGCAGCATATACTTCTTCACCTTATGTGCTATCTTCCTCTTC
	ABP2rbsF	ACAGTCATCGTGCACATAGTCTGCCTCCGTAAGGGAATTAGAAGAATGAGTGTGATCGCTAAAC
	ACPR	AGCCAACTCAGCTTCCTTTCGCTAGGCGACCACAGGTTTGCG
ABP2-ML06	ACPvecF	CGAAAGGAAGCTGAGTTGGCTGCT
	ABP2VR	AGACTATGTGCACGATGACTGTGAGGCAGCATATGTATATCTCCTTCTTAAAGTTAAACAAAATTATTTC
	ABP2P22F	ACAGTCATCGTGCACATAGTCTGCCTCCGATGAGTATTAAGAATTTCTTTGATGCTGCGC
	ACPR	AGCCAACTCAGCTTCCTTTCGCTAGGCGACCACAGGTTTGCG
ML06-StrepABD	rbsAmCPF	GGGAATTAGAAGAATGAGTGTGATCGCTAAACAAATGACCTAC
	FlsmapR	CACCAGAACCAGCTGCTGAACCTGCTGAACCATACTTCTTCACCTTATGTGCTATCTTCCTCTTC
	FlstrepADBF	GTTCAGCAGCTGGTTCTGGTGAGTTCATGGCTGAAGCTAAAGTTCTGGC
	rbsstrepADBR	CGATCACACTCATTCTTCTAATTCCCTTACGGCAGAGCAGCCAGGATTTC
StrepABD-ML06	FlAP22F	GTTCAGCAGCTGGTTCTGGTGAGTTCATGAGTATTAAGAATTTCTTTGATGCTGCGC
	VecR	CATATGTATATCTCCTTCTTAAAGTTAAACAAAATTATTTCTA
	VstrepABDF	ATTTTGTTTAACTTTAAGAAGGAGATATACATATGGCTGAAGCTAAAGTTCTGGCTAAC
	FlstrepABDR	CACCAGAACCAGCTGCTGAACCTGCTGAACCCGGCAGAGCAGCCAGGATTTC
ML06-HDD	rbsAmCPF	GGGAATTAGAAGAATGAGTGTGATCGCTAAACAAATGACCTAC
	FlsmapR	CACCAGAACCAGCTGCTGAACCTGCTGAACCATACTTCTTCACCTTATGTGCTATCTTCCTCTTC
	FlHDDF	GTTCAGCAGCTGGTTCTGGTGAGTTCATGAAACAGCTGGAAAAAGAACTGAAACAGCTGGA
	rbsHDDR	CGATCACACTCATTCTTCTAATTCCCTTACGCCTGCAGTTTTTTTTTCAGCTGCGC
HDD-ML06	FlAP22F	GTTCAGCAGCTGGTTCTGGTGAGTTCATGAGTATTAAGAATTTCTTTGATGCTGCGC
	VecR	CATATGTATATCTCCTTCTTAAAGTTAAACAAAATTATTTCTA
	VHDDF	ATTTTGTTTAACTTTAAGAAGGAGATATACATATGAAACAGCTGGAAAAAGAACTGAAACAGCTGGA
	FlHDDF	CACCAGAACCAGCTGCTGAACCTGCTGAACCCGCCTGCAGTTTTTTTTTCAGCTGCGC

Table S2. Bacterial strains used in the study, their source and antimicrobial resistance.

Strain	Source	Drug resistance
Acinetobacter baumannii 50-16	Hospital strain, intensive care unit	AMP, CHL
Acinetobacter baumannii 54-16		n/d
Acinetobacter baumannii 55-16		n/d
Acinetobacter baumannii 58-16		AMP, CHL, CTZ, CAZ, GEN, MEM
Acinetobacter baumannii ATCC 19606	Reference laboratory strain, ATCC collection	AMP, CHL, GEN

Klebsiella pneumoniae 104-14	Hospital strain, outpatient hospital	AMP, CHL, CTZ, CAZ, GEN; ETP, IPM, MEM
Klebsiella pneumoniae 08-15	Hospital strain, inpatient hospital	AMP, TET, CHL, CTZ, CAZ, MEM
Klebsiella pneumoniae 141-14		AMP, TET, CHL, CTZ, CAZ, GEN, MEM; CIP
Klebsiella pneumoniae 45-16		AMP, TET, CHL, CTZ, CAZ, GEN
Klebsiella pneumoniae ATCC 10031	Reference laboratory strain, ATCC collection	AMP
Pseudomonas aeruginosa 38-16	Hospital strain, intensive care unit	AMP, TET, CHL, CTZ, CAZ, GEN, MEM; CPZ/SUL
Pseudomonas aeruginosa 43-16		AMP, TET, CHL, CTZ, CAZ, MEM
Pseudomonas aeruginosa 21	Hospital strain, inpatient hospital	n/d
Pseudomonas aeruginosa ATCC 9027	Reference laboratory strain, ATCC collection	AMP, TET, CHL, CTZ
Pseudomonas aeruginosa ATCC 10145	Reference laboratory strain, ATCC collection	AMP, TET, CHL, CTZ
Escherichia coli 35-15	Hospital strain, inpatient hospital	n/d
Escherichia coli 65-1114		n/d
Escherichia coli 127-15		n/d
Escherichia coli ATCC 25922	Reference laboratory strain, ATCC collection	AMP
Escherichia coli ATCC 11229	Reference laboratory strain, ATCC collection	Sensitive

AMP – Ampicillin; CAZ – Ceftazidime; CHL – Chloramphenicol; CIP – Ciprofloxacin; CPZ/SUL – Cefoperazone/Sulbactam; CTX – Cefotaxime; CXM – Cefuroxime; ETP – Ertapenem; GEN – Gentamicin; IPM – Imipenem; MEM – Meropenem; PMB – Polymyxin B; TET – Tetracycline. n/d – no data.

Antibiotic susceptibility analysis of bacterial strains was performed by the broth microdilution method using Mueller-Hinton broth, according to ISO recommendations (ISO 20776-1. Clinical laboratory testing and in vitro diagnostic test systems—Susceptibility testing of infectious agents and evaluation of performance of antimicrobial susceptibility testing devices—part 1. Geneva, Switzerland: International Organization for Standardization, 2006). Results were interpreted according to The European Committee on Antimicrobial Susceptibility Testing. Breakpoint tables for interpretation of MICs and zone diameters. Version 15.0, 2025.
